# Towards a taxonomy of geodiversity

**DOI:** 10.1098/rsta.2023.0060

**Published:** 2024-04-01

**Authors:** Jan Hjort, Arie C. Seijmonsbergen, Julia Kemppinen, Helena Tukiainen, Tuija Maliniemi, John E. Gordon, Janne Alahuhta, Murray Gray

**Affiliations:** ^1^ Geography Research Unit, University of Oulu, Pentti Kaiteran katu 1, 90570 Oulu, Finland; ^2^ Institute for Biodiversity and Ecosystem Dynamics (IBED), University of Amsterdam, PO Box 94240, 1090GE Amsterdam, The Netherlands; ^3^ School of Geography and Sustainable Development,University of St Andrews, St Andrews KY16 9AL, UK; ^4^ School of Geography, Queen Mary University of London, Mile End Road, London E1 4NS, UK

**Keywords:** geodiversity, abiotic diversity, geofeature, hierarchical classification, taxonomy

## Abstract

Geodiversity is a topical concept in earth and environmental sciences. Geodiversity information is needed to conserve nature, use ecosystem services and achieve sustainable development goals. Despite the increasing demand for geodiversity data, there exists no comprehensive system for categorizing geodiversity. Here, we present a hierarchically structured taxonomy that is potentially applicable in mapping and quantifying geodiversity across different regions, environments and scales. In this taxonomy, the main components of geodiversity are geology, geomorphology, hydrology and pedology. We propose a six-level hierarchical system where the components of geodiversity are classified at progressively lower taxonomic levels based on their genesis, physical–chemical properties and morphology. This comprehensive taxonomy can be used to compile geodiversity information for scientific research and various applications of value to society and nature conservation. Ultimately, this hierarchical system is the first step towards developing a global geodiversity taxonomy.

This article is part of the Theo Murphy meeting issue ‘Geodiversity for science and society’.

## 1. Introduction

Geodiversity research is a rapidly developing field and a relatively new paradigm in earth and environmental sciences [1,2]. Geodiversity refers to the variability of abiotic features on the Earth's surface and in the subsurface. More precisely, it has often been defined as ‘the natural range (diversity) of geological (rocks, minerals, fossils), geomorphological (landforms, topography, physical processes), soil and hydrological features' [3,4]. Thus, in addition to commonly considered geological and geomorphological components, pedological (e.g. soil types and physical and chemical properties of soils) and hydrological features such as lakes, groundwater and snow are essential constituents of geodiversity [5–7].

Geodiversity information is required for several scientific and applied purposes (e.g. [2,8–11]). Both qualitative and quantitative data on geodiversity are needed in conservation actions [12–14], to sustain nature's services to people [15,16], to provide a sound basis for environmental management [17] and to achieve sustainable development goals [18–20]. A common shortcoming in previous (especially quantitative) geodiversity studies has been the omission of certain components of geodiversity or inconsistency of data, i.e. the included features have been acquired at different hierarchical level(s) [21,22]. For example, studies have often considered geological and geomorphological features but have omitted soils or hydrological features [21]. This can partly be explained by the lack of suitable data and/or the use of an alternative definition of geodiversity [22] but also by the absence of a classification system. Moreover, some components are observed at a general level (e.g. ‘a lake’ or ‘a river’ in hydrology), whereas specific rocks (e.g. diorite and quartzite) and landforms such as parabolic sand dune and river terrace are identified in more detail in geology and geomorphology [23]. Different categorical inconsistencies in data could bias the overall assessment of geodiversity and how certain components of geodiversity affect the studied subjects such as ecosystems and biodiversity.

Despite the substantial need for data and progress in the field, there is a lack of a comprehensive classification system for mapping and measuring geodiversity [21,22,24–26]. In comparison, such a hierarchical system is fundamental for exploring and managing biodiversity [27]. However, developing a taxonomy for geodiversity is not as straightforward as for biodiversity because most of the abiotic features are complex and lack evolutionary relationships (cf. phylogeny in biology). For this reason, currently there are few systems to categorize geodiversity [28] or objects comparable to the features of geodiversity (e.g. [29]) and they do not include all the components (geology, geomorphology, hydrology and pedology) nor explicitly consider hierarchical relations between specific features [30,31]. These deficiencies reduce the comparability of geodiversity studies [32], complicate the exploration of mechanistic links between biotic and abiotic nature [33] and may hamper geoconservation efforts and sustainable environmental management [17,34]. Consequently, the lack of geodiversity taxonomy may hinder the advancement of geodiversity science and its applications in, for example, climate change adaptation, biodiversity loss and sustainable development [13,19].

Here, we present a tentative taxonomy for geodiversity on the Earth's surface and in the subsurface (cf. Earth's ‘critical zone’, [35]). More precisely, we (i) provide a hierarchically structured taxonomy that can be used in observing and quantifying geodiversity; and (ii) explore the applicability of the taxonomy by classifying features of geodiversity mapped at a local and landscape scale. The focus is on the development of a taxonomy of geodiversity *per se*, and therefore, beyond the scope of this study are the definition and description (e.g. [36]), consideration of qualitative aspects (e.g. [12,34]), presentation of mapping methodologies [37–40] and quantification of features of geodiversity [41].

## 2. Geodiversity taxonomy

**Figure 1.**
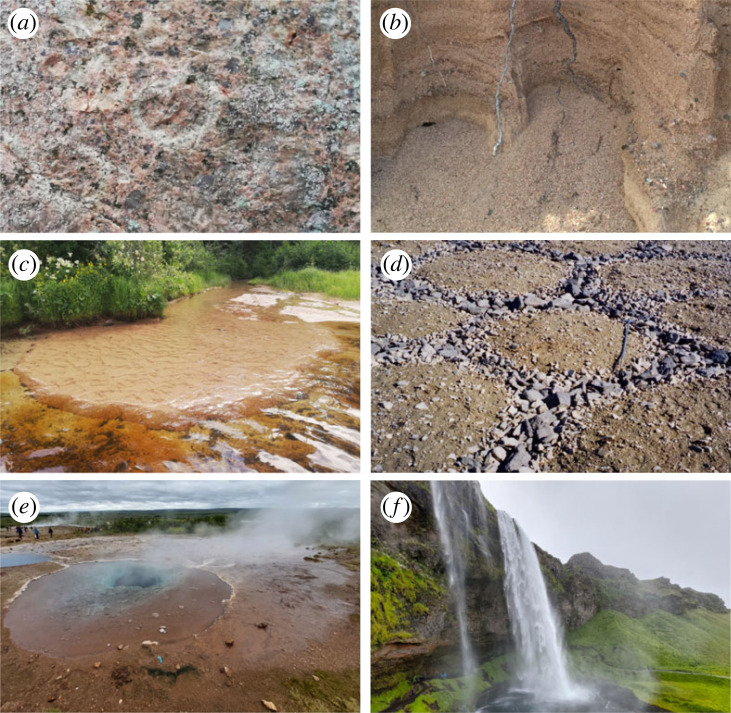
Examples of geofeatures from the geological (*a*,*b*), geomorphological (*c*,*d*) and hydrological (*e*,*f*) components of geodiversity. (*a*) A close-up of granite, (*b*) a layered sand deposit, (*c*) a small delta, (*d*) periglacial patterned ground, (*e*) a spring pool and (*f*) waterfalls. (Online version in colour.)

To develop a simple, adaptable and transferable system for classifying geodiversity, we focus on geofeatures that are speciﬁc to geology, geomorphology, pedology and hydrology, analogous to the elements of geodiversity (*sensu* [42]) (figure 1). Geofeatures are relatively clearly defined objects of geodiversity [5,40], easier to observe than complex measures of abiotic diversity [22,41] and have been the focus of land use planning and conservation actions [42].

We propose a six-level hierarchical classification system. The *components of geodiversity* (geology, geomorphology, hydrology and pedology i.e. soils; [3]) formed the first taxonomic level in the developed hierarchical system (table 1). At levels 2–6, geofeatures are classified based on their genesis, physical–chemical properties and morphology. The system does not include dynamic processes *per se* but indicators of processes. For example, the aim is not to map the type, activity or force of a process (e.g. tectonic activity or turbulent stream flow), but rather the focus is on geological structures and landforms originated by tectonic activity and features indicating turbulent water flow. However, processes are integral across levels 2–6 in the classification (see electronic supplementary material, S1). At the second level, the components were subdivided into nine *classes of geofeatures*. Of the components, geology, pedology and hydrology included two classes and geomorphology, three classes. The third hierarchical level included 33 *groups of geofeatures* and the fourth level 118 *subgroups of geofeatures* (electronic supplementary material, S1). At the fifth taxonomic level are *geofeatures* (i.e. specific elements of geodiversity), which were divided into *subtypes of geofeatures* at the sixth taxonomical level. We estimate that the fifth taxonomic level contains some thousands of different geofeatures and the sixth taxonomic level tens of thousands of subtypes of geofeatures (e.g. [43–45]). However, if fossils are observed at the species level, there exist up to 300 000 subtypes of geofeatures just in this category [46]. Hence at levels 5 and 6 in electronic supplementary material, S1, we have not attempted a comprehensive listing and only indicative examples of geofeatures and subtypes of geofeatures are presented.

**Figure 2.**
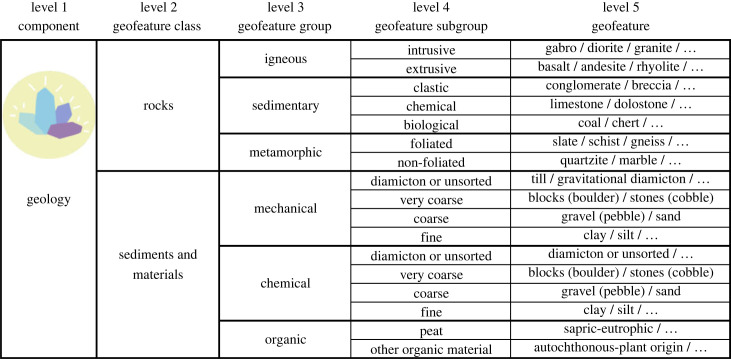
Classification of the geology component with selected examples of geofeatures (separated by /). Note that most of the geofeature lists (…) are not exhaustive (see electronic supplementary material S1 for more examples of geofeatures). Subtypes of geofeatures are at level 6, but they are not listed in this general representation of the classification. (Online version in colour.)

**Table 1.** The hierarchical classification system of geodiversity with examples from the geological (figure 1*a*), geomorphological (figure 1*c*) and hydrological (figure 1*e*) components.

level	name of category	example figure 1*a*	example figure 1*c*	example figure 1*e*
1	component of geodiversity	geology	geomorphology	hydrology
2	class of geofeature	rocks	exogenic	surface water
3	group of geofeature	igneous	deposition	spring
4	subgroup of geofeature	intrusive	fluvial–alluvial	perennial
5	geofeature	granite	delta	pool
6	subtype of geofeature	rapakivi granite	river-dominated	thermal

In the development of the taxonomy, we consulted comparable hierarchical systems [28,29,47], geoscientific textbooks (e.g. [48–53]), benchmark compilations (e.g. [43,44,54]) and journal articles (e.g. [55–58]). Some of the classes (level 2, e.g. rocks; [48,59]), groups (level 3, e.g. soil types; [60]) and subgroups (level 4, e.g. mass movements; [58]) of geofeatures followed the systems presented in the literature but many of them (e.g. subcategories of geomorphology and hydrology) were revised considering the purpose of the geodiversity taxonomy (i.e. the revised categories were developed for this study and did not follow a specific reference or system).

Under the geological component, the two classes were rocks, and sediments and materials. Rocks represent consolidated (solid) and sediments unconsolidated (loose) material (e.g. [61]). Rocks were further classified at levels 3 (three categories) and 4 (seven categories) based on their process of formation and geological setting (figure 2). For example, the subdivision of igneous rocks to intrusive and extrusive is a more accessible approach than, for example, the chemistry-based classification to felsic, intermediate, mafic and ultramafic rocks [48,59,62]. Mechanical and chemical sediments were classified based on granulometry (particle size), but organic materials were divided first to peat and other organic material, and then based on the level of decomposition, nutrients and/or the origin of the organic material (e.g. [49,61,63,64]) (electronic supplementary material, S1). Although minerals and fossils are central in geodiversity [3], they were considered as subtypes of geofeatures (level 6) because rocks and most of the sediments are composed of minerals, and fossils occur in specific (mostly sedimentary) rocks [44,54].

**Figure 3.**
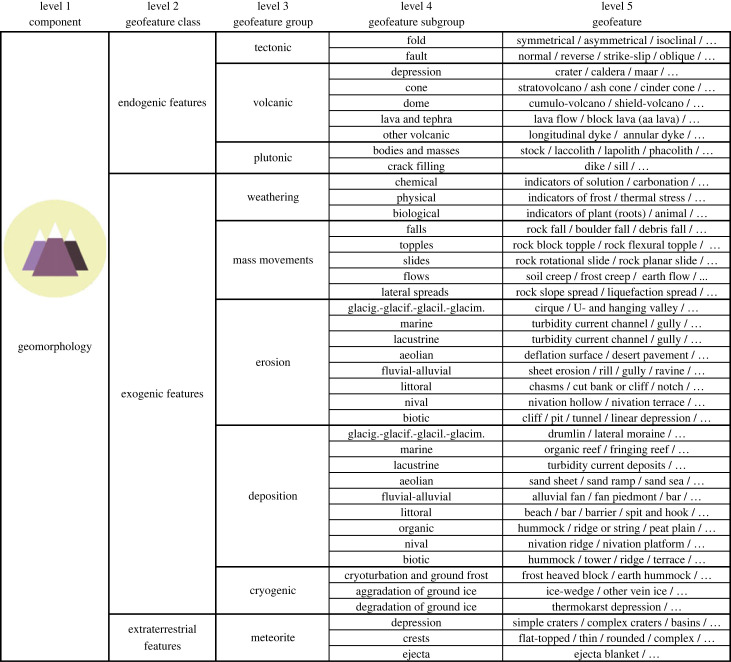
Classification of the geomorphology component with selected examples of geofeatures (separated by /). Note that the geofeature lists (…) are not exhaustive (see electronic supplementary material S1 for more examples of geofeatures). Subtypes of geofeatures are at level 6, but they are not listed in this general representation of the classification. Glacig.-glacif.-glacil.-glacim. = Glacigenic-glacifluvial-glacilacustrine-glacimarine. (Online version in colour.)

Under the geomorphological component, classes of geofeatures were endogenic, exogenic and extraterrestrial (figure 3; [43]). They were mainly subdivided based on the genesis of geofeatures at levels 3 (nine categories) and 4 (40 categories) [43,47,53]. For example, the exogenic class contained cryogenic features (level 3), which included cryoturbation and ground frost (level 4) and patterned ground (level 5; figure 1*d*). Examples of subtypes of geofeatures (level 6) were not presented but, for instance, patterned ground could be further divided into sorted and non-sorted circles, polygons, nets, steps and stripes [65]. In the developed system, topography was an inherent part of geomorphology and was not presented separately in the main taxonomy (electronic supplementary material, S1). However, topographical features could be mapped separately at local scales (e.g. using visual observation [66] or light detection and ranging technology) where there is little or no variation in geomorphological geofeatures (electronic supplementary material, S2). Moreover, if geomorphology cannot be mapped or there are no geomorphological data available, digital elevation model-based topographical features could supplement or substitute geomorphological geofeatures in regional or global scale studies (e.g. [5,57]).

**Figure 4.**
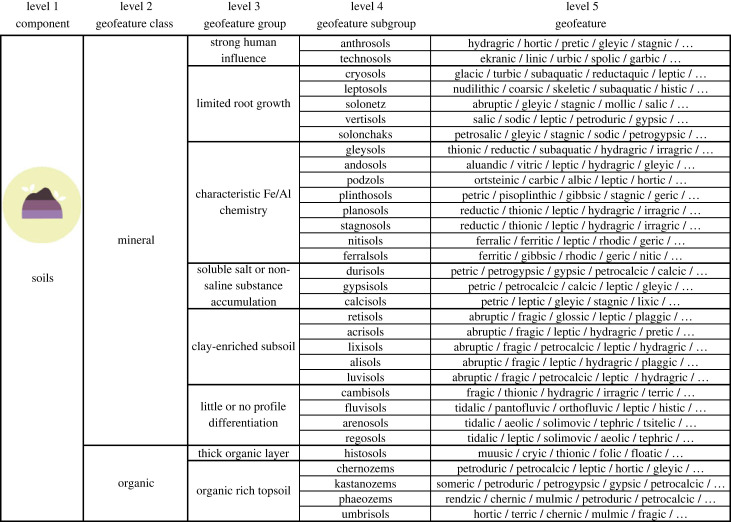
Classification of the soil component with selected examples of geofeatures (separated by /). Note that the geofeature names refer to the principal qualifiers [60] and lists (…) are not exhaustive (see electronic supplementary material S1). Subtypes of geofeatures are at level 6, but they are not listed in this general representation of the classification. (Online version in colour.)

Under the soil component, we used the international soil classification system of the International Union of Soil Sciences [60]. Two main classes (mineral and organic soils) were followed by eight categories at level 3, which were based on the soil-forming factors or processes that most clearly condition the soil (e.g. characteristic Fe/Al chemistry and thick organic layer) (figure 4). Geofeatures and subtypes of geofeatures can be defined based on principal and supplementary qualifiers of soils (see [60]; electronic supplementary material, S1).

**Figure 5.**
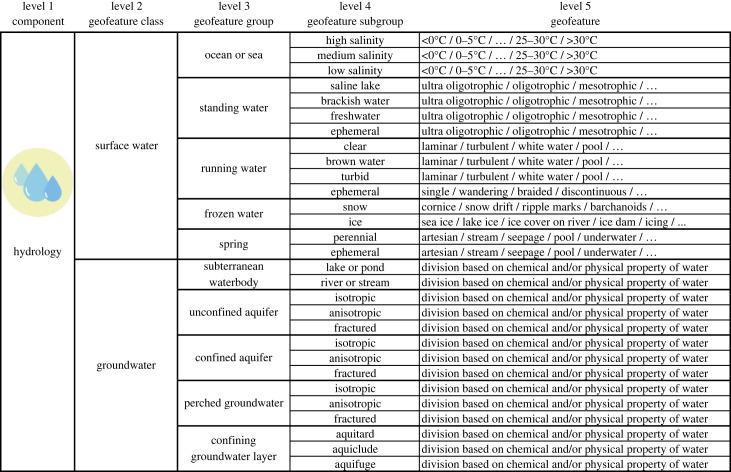
Classification of the hydrology component with selected examples of geofeatures (separated by /). Note that the geofeature lists (…) are not exhaustive (see electronic supplementary material S1 for more examples of geofeatures). Subtypes of geofeatures are at level 6, but they are not listed in this general representation of the classification. (Online version in colour.)

Under the hydrological component, surface water and groundwater were the two logical classes (figure 5; [67]). Surface water contained the groups of ocean or sea, standing water, running water, frozen water and spring. Geofeatures in the group of standing water were categorized mainly based on the salinity and nutrient level of the water at levels 4 and 5, respectively [51,67–69]. For running waters, the properties of water (colour) and flow type were central [55,70–73]. The group of frozen water had two subgroups (snow and ice) with a relatively large number of potential geofeatures at level 5 [56,74–77]. The group of spring and related geofeatures was included in the class of surface water as surficial manifestations of groundwater, whereas subsurface geofeatures were included in the class of groundwater [78–80] (figure 5; electronic supplementary material, S1).

## 3. Applying the geodiversity taxonomy

Geofeatures form the basis of our taxonomy and they can be measured in multiple ways. In addition to a simple presence–absence scale (e.g. [5,81,82]), geofeatures can be quantified by measuring their properties (e.g. size, composition, physical–chemical characteristics, activity and age) or qualitatively by assessing geofeatures' value(s) [37,41,83]. For example, Gray [84] listed a total of 31 specific values of geodiversity that could be assessed and quantified (see also [12]). Moreover, mapped geofeatures could be reclassified to functional groups based on their physical, chemical, morphological and/or temporal properties ([6,38,39]; cf. traits and functional groups in ecology; e.g. [85]). Further, depending on research aims and scale of study, geofeatures and their indicators could also be combined for mapping of landform assemblages, process domains and landsystems, incorporating spatial and temporal aspects (e.g. [50,86,87]).

We tested the geodiversity classification system by using two applicable datasets with observations of geofeatures at a local (circular areas with a 5 m radius) and landscape (500 × 500 m grid cells) scale (see electronic supplementary material, S3). The aim was to provide an indicative assessment of the performance of taxonomy in classifying pre-mapped field-based observations from high-latitude environments and, on the other hand, how the taxonomy may support the compilation of original geodiversity data. Moreover, we explored how a simple measure of geodiversity (here georichness; [40]) varied at different taxonomic levels and correlated between them (electronic supplementary material, S3). It should be noted that soils were not included in the datasets and geomorphology was supplemented by topographical geofeatures in the local-scale data.

In both datasets, it was possible with some restrictions to classify observations up to level 5 (geofeatures) (electronic supplementary material, table S1). At the landscape scale, most of the geomorphological features could have been classified to level 6. In general, the classification of geofeatures was relatively straightforward but there were also restrictions owing to the deficiencies in the source data. For example, sediments (e.g. organic material) and hydrological features were not originally mapped with sufficient details. Hydrological features were mostly considered at higher taxonomic levels (thematic accuracy fitted at best to level 3 or 4) when compared to the features of geology and geomorphology (most of the features were mapped at level 5 or 6). Explorations with the empirical test data showed that information acquired at a general level (e.g. at level 3) may well characterize geodiversity at a more detailed level (electronic supplementary material, table S2). Although our test data represented high-latitude environments and focused on specific components of geodiversity (especially geology and geomorphology), group (level 3) and subgroup (level 4) observations may illustrate overall variation of geofeatures surprisingly well (electronic supplementary material, table S2).

The successful tests of our taxonomy with the two field-based datasets opens possibilities to select specific scales of observations and measurements. For example, it may be challenging to observe all the potential geofeatures or subtypes of geofeatures from an area (electronic supplementary material, S1). At the higher taxonomic levels (3 and 4), most of the geofeatures should be observable in the field without specific instruments or laboratory tests and with a reasonable amount of geoscientific training. Depending on the considered component of geodiversity, the acquisition of data at lower levels (5 and 6; table 1) requires subject-specific knowledge and training. However, it should be possible to identify most of the geological, geomorphological and hydrological features with basic researcher training and field experience at the fifth level (figures 2 and 3). Naturally, some of the geofeatures (e.g. soils and geofeatures of groundwater) require more comprehensive knowledge and/or need experience with handling measurement equipment (figures 4 and 5). Specific information for the identification of geofeatures and subtypes of geofeatures can be acquired using sampling (e.g. soil, sediment or water), measuring (e.g. field meters and probes for hydrology) and drone imaging (e.g. hyperspectral imaging for rock or mineral detection).

Spatial scale can be a challenge in observing geodiversity (cf. [26,28,29]). With the proposed hierarchical taxonomy, we introduced flexibility and freedom for researchers in different environments to select their desired spatial scale, their components of geodiversity, and hierarchical level(s), depending on their aim and application of the study. For example, it may be more feasible to observe geofeatures at the subgroup level or exclude certain features (e.g. soils and groundwater) at the landscape and regional-scale analyses. It may be enough to use group or subgroup of geofeatures when investigating, for instance, climate change mitigation effects of geodiversity at broad scales [13,88]. More detailed taxonomy (level 5 or 6) is likely needed in studies on local-scale geodiversity–biodiversity relationships (e.g. [89]), especially if the aim is to reveal mechanistic processes, not just patterns [33]. Naturally, omissions affect the comprehensive exploration of geodiversity but targeting the focus according to the aims (and mapping skills) can be a practical solution in regional-scale studies [22,90]. Moreover, the taxonomy enables hierarchical upscaling of geofeatures and subtypes of geofeatures (e.g. observations at level 5 can be upscaled to level 4 or 3).

## 4. Next steps in developing the geodiversity taxonomy

We designed our hierarchical taxonomy after reviewing and reorganizing existing classifications from geology, geomorphology, pedology and hydrology, and to optimize the system for geodiversity observations and measurements at various levels of detail. The taxonomy was tested using two existing geodiversity datasets, collected in a high-latitude environment at a local and a landscape scale. The results suggest that, with minor modifications, consistent and quantitative geodiversity information at different levels can be collected (table 1, electronic supplementary material, tables S1 and S2).

This taxonomy is regarded as a first step towards an operational observation and mapping system across different environments. Owing to the fact that the taxonomy covers all the components of geodiversity and features across spatial scales (e.g. from minerals to folded mountain ranges; electronic supplementary material, S1), it should be widely applicable (cf. [28,29]). However, the taxonomy is open to improvements related to the observed geofeatures and structure of the system. For example, the taxonomy lacks certain dynamic features and processes, which can be challenging to observe. Under the geomorphology component, the system could be enriched with transport processes at the group level (level 3 of the hierarchy). Transport processes were not included because they were considered indirectly in the groups of erosion and deposition (i.e. there cannot exist erosion or deposition without the transportation of material). Indicators of erosion and deposition can be easier to detect compared to features indicating transportation that may have occurred a considerable time ago (e.g. wind ripples or glacial striae). Moreover, the role of topography and topographical features could be reassessed in future studies [91]. Here, geomorphons were simple and suitable features to characterize the basic elements of topography [57,66]. However, the applicability of other classifications of topography or geomorphometric indexes should be explored [92,93].

The developed taxonomy may require new categories to account for geofeatures’ intrinsic heterogeneity (e.g. [29,52]) because geofeatures can have complex genesis, material and/or structure and ages [8,94,95]. An intrinsic property of the system is that it includes, to some extent, double or triple counting. For example, organic material is a factor in geology (sediments and materials), geomorphology (exogenic geofeatures) and soils to ensure the comprehensiveness of individual geodiversity components. The issue of multiple counting could be managed by modifying the taxonomy or excluding problematic geofeatures when collecting or using the data. In the end, one should keep the purpose of use in mind when employing or applying the taxonomy.

The tentative nature of the present taxonomy calls for further development of the system by geodiversity researchers in collaboration with experts in geology (petrology and mineralogy), geomorphology, soil science (pedology) and hydrology. Special attention could be given to the usability of the applied soil system [60] and the classification of subsurface [96,97] and hydrological geofeatures. For example, hydrological features are central but often neglected in geodiversity studies [22,28]. Thus, there is a lack of well-established systems for the hierarchical classification of hydrological geofeatures. The classification of water bodies, snow, ice and groundwater can be based on different physical, chemical, morphological and temporal properties, and it may be challenging to develop a global system (e.g. [98–101]). The same challenges are common also for other components of geodiversity and the most suitable classification system can be context dependent. Despite the challenges in the development of a global taxonomy of geodiversity we consider this goal worth pursuing and the presented system an essential and required step forward. Moreover, an online system with a comprehensive list and definitions of geofeatures similar to that of, for example, the Common International Classification of Ecosystem Services (https://cices.eu/) is an important objective in the future.

## 5. Conclusion

In this study, we presented and tested a tentative hierarchical taxonomy of geodiversity that could be used for classification, inventory and analytical purposes. The basic elements of the hierarchical system are geofeatures, which can be grouped or refined in higher or lower taxonomic levels in practice. We found that the developed hierarchical system facilitates consistent geodiversity mapping and classification of geodiversity information from local to regional scales and consider that further development of the system requires multidisciplinary collaboration between (geo)scientists with expertise in a variety of environments. Remaining challenges include refining the basic classification of geofeatures (mainly levels 4–6) and consideration of the dynamic, complex and qualitative aspects of geodiversity. A comprehensive and hierarchically sound taxonomy benefits the field of geodiversity and promotes the use of geodiversity information more widely in different scientific, societal and nature conservation applications.

## Data Availability

The data are provided in electronic supplementary material [102].
